# Are trichomes involved in the biomechanical systems of *Cucurbita* leaf petioles?

**DOI:** 10.1007/s00425-015-2388-z

**Published:** 2015-08-26

**Authors:** Urszula Zajączkowska, Stanisław Kucharski, Dominika Guzek

**Affiliations:** Department of Forest Botany, Faculty of Forestry, Warsaw University of Life Sciences, 159 Nowoursynowska St., 02-776 Warsaw, Poland; Department of Mechanics of Materials, Institute of Fundamental Technological Research, Polish Academy of Sciences, 5B Pawińskiego St., 02-106 Warsaw, Poland; Division of Engineering in Nutrition, Warsaw University of Life Sciences, 159c Nowoursynowska St., 02-776 Warsaw, Poland

**Keywords:** Collenchyma, Epidermis, Plant biomechanics, Shape optimization, Tropic response

## Abstract

**Trichomes are involved in petiole movement and likely function as a part of the plant biomechanical system serving as an additional reservoir of hydrostatic pressure.**

The large, non-glandular trichomes on *Cucurbita* petioles occur across collenchyma strands. Time-lapse imaging was used to study the leaf reorientation of *Cucurbita maxima* ‘Bambino’ plants placed in horizontal position. The experiment comprised four variants of the large non-glandular petiole trichomes: (1) intact, (2) mechanically removed, (3) dehydrated, and (4) intact but with longitudinally injured petioles. Isolated strands of collenchyma with intact epidermis or epidermis mechanically removed from the abaxial and adaxial sides of the petiole were subjected to breaking test. The stiffness of the non-isolated tissue with intact epidermis was measured using the micro-indentation method. Petioles without trichomes did not exhibit tropic response, and the dehydration of trichomes slowed and prevented complete leaf reorientation. Isolated strands of collenchyma showed no correlation between strength values and position on the petiole. However, strands of collenchyma with epidermis exhibited a significantly greater strength regardless of their position on the petiole. The indentation test showed that non-isolated collenchyma is stiffer on the abaxial side of the petiole. Trichomes from the abaxial side of the petiole were larger at their base. The application of the ‘tensile triangles method’ revealed that these trichomes had a biomechanically optimized shape in comparison to the adaxial side. We conclude that trichomes can be involved in plant biomechanical system and serve as an additional reservoir of hydrostatic pressure that is necessary for maintaining petioles in the prestressed state.

**Electronic supplementary material:**

The online version of this article (doi:10.1007/s00425-015-2388-z) contains supplementary material, which is available to authorized users.

## Introduction

In herbaceous plants, tissue stress (TS) is a result of the interactions between anatomical structures, mainly cell walls, and physiological processes related to the osmotic conditions of the cell. TS is an important element that keeps the tissues, organs, and entire body at high level of potential energy (Niklas 1992; Hejnowicz 2011). Due to the existence of TS and the state of pre-stress, the amount of energy necessary for plant movement reactions can be lowered (Hejnowicz 1997; Spatz et al. 1999; Elices 2000; Vincent 2012). Therefore, during water stress, which indicates a decrease in TS, the plant is not able to perform any movements (Esmon et al. 2004).

There are no reports on the biomechanical significance of petiole trichomes, although the epidermis is the important structure involved in developing TS in growing non-lignified organs in herbaceous plants (Niklas and Paolillo 1997; Domínguez et al. 2011; Vincent 2012). Trichomes are outgrowths of protodermal origin that can exist in all morphological parts of a plant (Fahn 2000; Werker 2000; Evert 2006). They take on various morphological forms, from unicellular outgrowths to multicellular and branched structures (Arthur 1881; Ramaley 1902; Carlquist 1958; Wagner 1991; Bauer et al. 2011; Kraehmer and Baur 2013). The known functions of trichomes can serve to divide them into two main groups: secretory (glandular) and non-secretory (non-glandular) trichomes. The active substances in secretory trichomes exhibit activities similar to those of insecticides (Peiffer et al. 2009; Tian et al. 2012) and herbicides (Duke 1994). Non-secretory trichomes reduce transpiration (Wiegand 1910; Savé et al. 2000; Bacelar et al. 2004), act as hydrophobic layers for water droplets (Pierce et al. 2001), help plants survive undercooling (Harvey 1919), reflect light (Pierce et al. 2001; Levizou et al. 2005) and act as a mechanical barrier against insects (Agrawal 1998; Dalin and Björkman 2002; Sletvold et al. 2010).

**Fig. 1:**
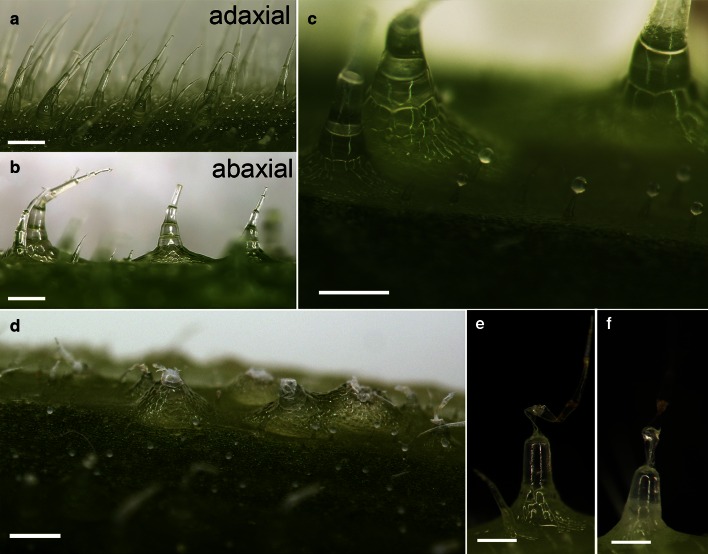
Morphology of the trichomes from the adaxial (**a**) and abaxial (**b**) petiole sides in *C. maxima* ‘Bambino’. Surface of the petioles before (**c**) and after (**d**) removing the apical part of trichomes. **e**–**f** Two examples of deformed trichomes after treatment with 7 % NaCl solution. *Scale bars* 500 µm

Petioles of *C. maxima* ‘Bambino’ are densely covered with hairs of both non-glandular and glandular trichomes. Glandular trichomes are very small with a maximum height (*h*_max_) of about 500 µm, topped with a spherical gland and evenly distributed over the entire surface of the petiole; smaller non-glandular trichomes have a similar size and distribution. However, larger non-glandular multicellular trichomes (*h*_max_ of approximately 3 mm), which can be seen with the naked eye, occur only on the strands of collenchyma (Fig. 1a, b). In this paper, the term “trichomes” will be used only for this latter type of trichome.

The petioles of winter squash are the active structures in the reorientation reactions of growing leaves. Additionally, petioles are characterized by a very simple anatomy with hollow tubes where the parenchyma constitutes the main ground tissue with bicollateral bundles arranged in rings and strands of angular collenchyma located above them. The petiole trichomes exist on protruding longitudinal collenchyma strands extending over the entire length of the petiole. Angular collenchyma is a living strengthening tissue characterized by cells that form irregular and non-lignified cell wall thickenings, frequently occurring in growing, non-lignified organs. Interestingly, the thickness of parenchyma cell wall changes with respect to the availability of water. The collenchymatous cell walls thicken probably due to pectins and shrink due to dehydration. This was shown by observations of anhydrous collenchyma, which resembles parenchyma (Hejnowicz and Barthlott 2005). Taking into account the fact that water is not able to transfer tensile stress in the cell wall (Hejnowicz 2011), it may be considered a potential counterhypothesis that this type of modification to the cell wall increases its ability to withstand tension. It is also believed that the collenchyma controls its mechanical efficiency to a certain extent by changes in turgor (Hejnowicz and Barthlott 2005; Schopfer 2006; Leroux 2012); therefore, reduced hydrostatic pressure in the cells results in reduced stiffness of collenchyma (Caliaro et al. 2013). On the other hand, some authors have suggested that collenchyma, which maintains strong tissue tension during growth, is not a hydrostatically active tissue due to the fact that changes in turgor do not control collenchyma action (Spatz et al. 1998; Speck et al. 1998).

The purpose of this study was to determine whether trichomes associated with collenchyma in petioles are able to affect the potential reorientation of the leaf. In other words, we investigated whether unknown biomechanical structures supporting the function of the collenchyma exist.

## Materials and methods

### Experimental

**Fig. 2:**
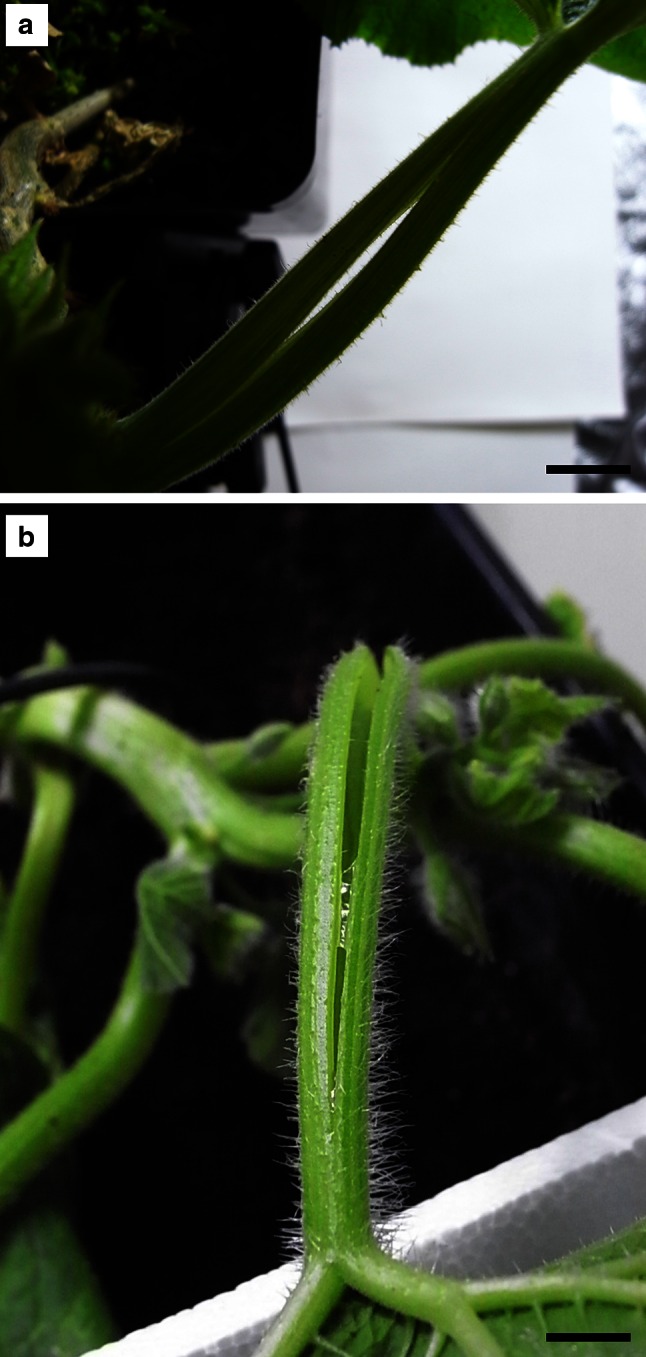
Photographs of a longitudinally cut petioles of *C. maxima* ‘Bambino’ (**a**–**b**). *Scale bar* 10 mm

Forty winter squash plants (*C. maxima* Duch. ‘Bambino’), aged 4–8 weeks and grown in the plant grow box from seeds (Vilmorin, Poland) under continuous light source (HPS Phytolite 600 W lamp, photon flux 1045 µmol m^−2^ s^−1^, luminous flux 100 klm) at 21 °C, were subjected to tropic reactions of young leaves with actively growing petioles. The experiment comprised four variants of trichomes: (1) intact, (2) mechanically removed, (3) dehydrated (Fig. 1c–f), and (iv) intact but with longitudinally injured petioles (Fig. 2a–d).

Trichomes were excised from the entire length of petioles (1–3 leaves from one plant) using a manual shaver. They were detached without causing injury to their multicellular base or other cells of the epidermis. In variant (3), the dehydration of the trichomes was based on the application of drops of a 7 % sodium chloride solution in 30-min intervals over the course of 6 h. The salt solution was prevented from coming into contact with the leaf epidermis. In variant (4), the wounds constituted longitudinal cuts on the adaxial (upper) and abaxial (lower) sides of the petiole along its entire length and throughout its thickness, i.e., up to the air channel.

To induce a tropic reaction, the plant in the pot was placed in the plant grow box perpendicular to gravity and the continuous light source.

### Time-lapse imaging

All plant movements were recorded using a Canon 5D Mark II digital camera with an external intervalometer (Aputure Timer Remote Cord) and Ricoh GX 200 with a built-in intervalometer. The intervals between frames were 15 or 30 min. The recording time ranged from 3 to 24 days. The study was performed from January 2014 through April 2015, using 21 plants; nine of these plants were recorded in time-lapse mode.

The resulting images were assembled into a movie using time-lapse software (Microprojects Prospect, Nova Scotia). Next, we conducted an analysis using the Tracker application (https://www.cabrillo.edu/~dbrown/tracker/) based on the Open Source Physics (OSP) Java framework and designed for kinetic analysis of video objects.

In variants (1) and (2), the reorientation of the leaf was analyzed with the software in the axis parallel to the gravity vector. In variants (3) and (4), only an observation was made.

### Microscopic observations

Morphology of the trichomes and petioles was observed using a scanning electron microscope (FEI QUANTA 200) at 25 kV and a stereoscopic microscope (Nikon SMZ 1000) integrated with NIS-Elements F software. Based on images of the trichomes, we analyzed their geometry using deltaCAD software in accordance with the ‘method of tensile triangles’ (Mattheck et al. 2007). This method is a graphic tool serving for assessing the optimal shape of the structures in light of minimizing the local stress concentration on the basis of observations from nature as well as computer models of stress distribution based on the finite element method.

An anatomical analysis of the strands of angular collenchyma was conducted for the central part of 11 petioles. Cross-sections of the analyzed parts were prepared for four collenchyma strands from the adaxial (upper) side and four on the abaxial (lower) side. Samples were collected by manual cutting and stained with a 1 % solution of safranin. An Olympus BX-61 microscope and the Cell^P software were used to conduct observation and analysis.

### Mechanical tests

#### Breaking stress of collenchyma with epidermis strands

**Fig. 3:**
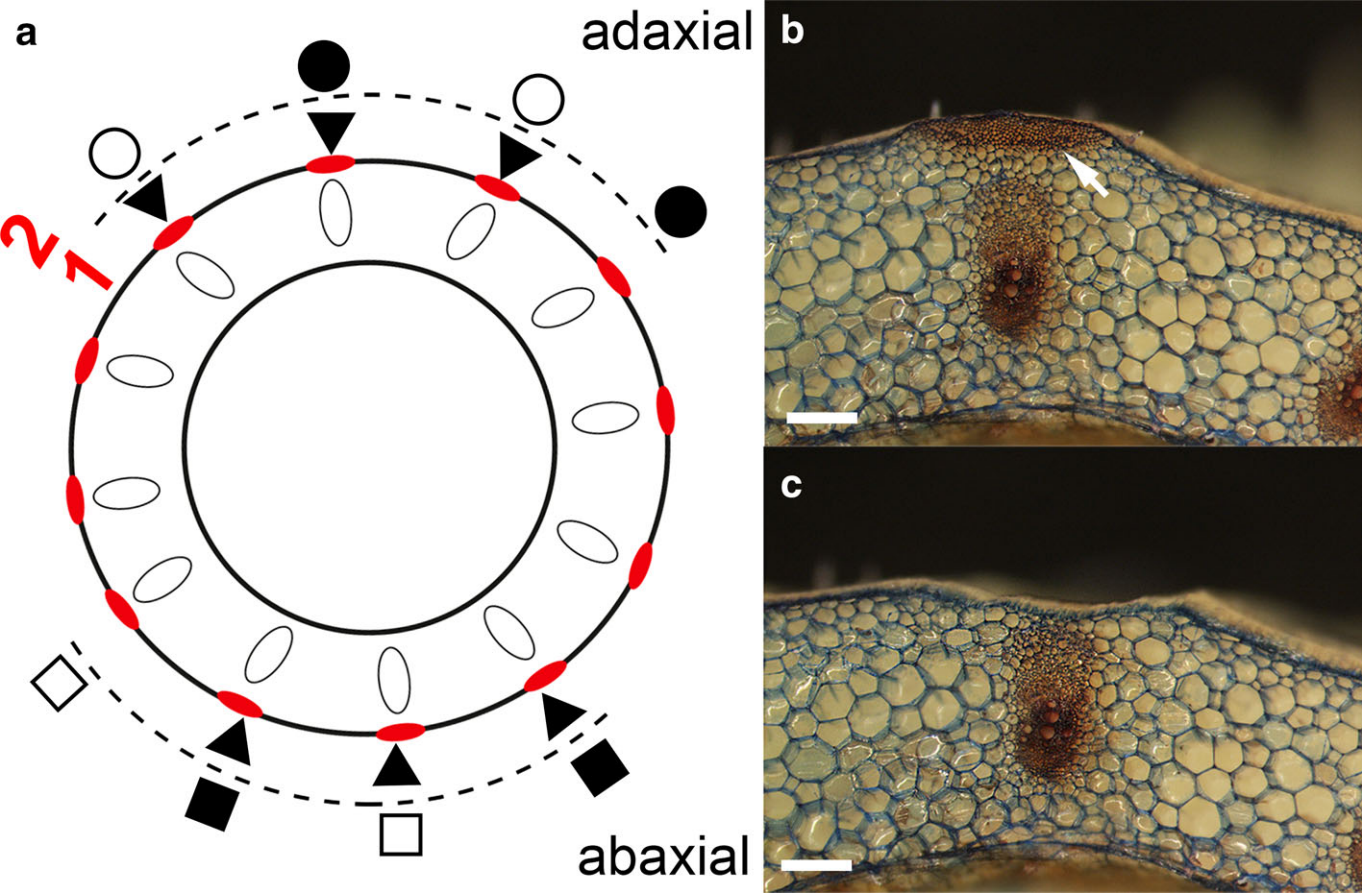
**a** Schematic cross-section view of the petiole subjected to breaking stress analysis. Variant 1—six strands of collenchyma with epidermis (*black triangles*). Variant 2—four strands from the adaxial (*circles*) and four strands from the abaxial (*squares*) sides of the petiole; strands of collenchyma with epidermis (*black circles* or *squares*) and without epidermis (*white circles* or *squares*). Fragment of the petiole cross-section before (**b**) and after (**c**) isolation of the collenchyma with epidermis strand (*white* *arrow*). The sections were stained with *safranin*–*alcian* *blue*. *Scale bars* 500 µm

We performed mechanical breaking stress tests of collenchyma strands from 18 leaf petioles using an Instron 5965 Universal Testing Machine (Model 5965; Instron Engineering Corp., Canton, MA, USA) for three variants (Fig. 3a–c): (1) strands of collenchyma with an epidermis layer together with trichomes (seven leaves with three strands on the adaxial side and three on the abaxial side of each petiole). (2) Strands of collenchyma with mechanically removed epidermis (six leaves with two strands with epidermis and two strands without epidermis on the adaxial and abaxial sides of the petiole). (3) Strands of collenchyma like in variant (2) but exposed surfaces of the samples were coated with vaseline to stop potential loss of turgor during the test (five leaves with two strands with epidermis and two strands without epidermis on the adaxial and abaxial sides of the petiole).

Before sampling, the cross-section of each petiole was scanned with a scanner (Epson Perfection V750 pro) with 1200 dpi resolution to measure cross-section areas of the collenchyma with the epidermis strand; this analysis was performed using ImageJ software (Schneider et al. 2012).

Each sample was tested immediately after collection with tweezers from the middle part of the petiole from a plant maintained in full turgor. In the first variant, measurements for 40 samples were recorded (two strands, one on each side, were too short and could not be tested); in the second variant, one strand could not be analyzed.

The epidermis was removed manually using a shaver and calipers. Because of the fact that epidermis in petioles has a thin cell wall and is strongly integrated with collenchyma, it was possible to remove epidermis from only 50–75 % of petiole surfaces.

The analysis was performed using a tension static test with mechanical vice grips. A crosshead speed of 1 mm s^−1^ was used. Total measurement time from sample collection to the end of the test was about 2–3 min. During this time, sample exhibited no signs of dehydration. The grip section, where the load frame holds the sample, was secured with an adhesive material to prevent sample slipping. The gauge length of the sample was 3 cm and the length of all collenchyma strands was 10 cm. The tests were carried out at 23 °C. Maximal values for the breaking load and displacement at the maximum load were calculated using Bluehill software.

### Indentation

**Fig. 4:**
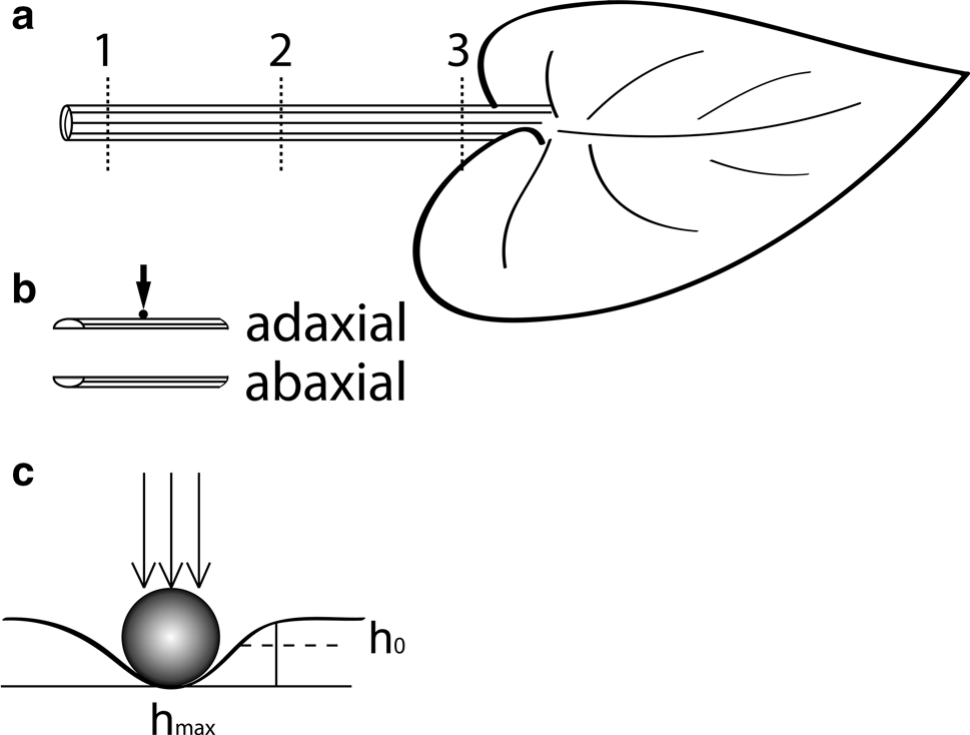
Scheme of indentation measurement. **a** Three locations on the petiole from which the samples were collected. **b** The samples from the adaxial and abaxial sides of the petiole. **c** Scheme of the indenter operation, where *h*_max_ is the maximum deformation, and *h*_0_ is the deformation remaining after removal of the indenter

Indentation tests were carried out on the original experimental setup, designed and constructed at the Institute of Fundamental Technological Research of the Polish Academy of Sciences (Kucharski and Mróz 2007; Levintant-Zayonts and Kucharski 2009). A spherical indenter tip (tungsten carbide) with a radius of 0.5 mm was used. The analysis was performed using five leaves for testing the stiffness of the collenchyma (with epidermis) strand, which was not isolated from the neighboring tissues (ground parenchyma and bicollateral vascular bundles). The samples were originated from three petiole locations: from the base of the petiole, from the middle part, and from the site right below the leaf blade (Fig. 4). In the case of all samples (10 mm in length by 5 mm in width), adaxial and abaxial sides of the petiole were tested.

The applied load and penetration depth of an indenter into the specimen were continuously measured, and analysis of the generated load-penetration (*P*–*h*) curve was used to obtain the mechanical properties of the indented materials. The load was measured at the base of the strain gauge bridge and optical sensors were used to estimate the penetration depth. Since the tip dimension was greater than the dimension of the individual cells, the registered *P*–*h* curves corresponded to the deformation of many cells and only the average cell properties were specified. The classic analysis of *P*–*h* curves, such as the Oliver–Pharr method (Oliver and Pharr 2004) could not be applied, since the constitutive laws describing the strain–stress state of the cells were unknown. As a result, the registered *P*–*h* curves were used only for a comparative study of the material response in different locations of the plant, and the *P*–*h* curves were considered to be compatible with an average internal pressure in the cells. Local and average inclinations were distinguished on the *P*–*h* curves and estimated as *S*_loc_ = d*P*/d*h* and *S*_av_ = *P*_max_/*h*_max_, respectively.

## Results

### Time-lapse imaging

**Fig. 5:**
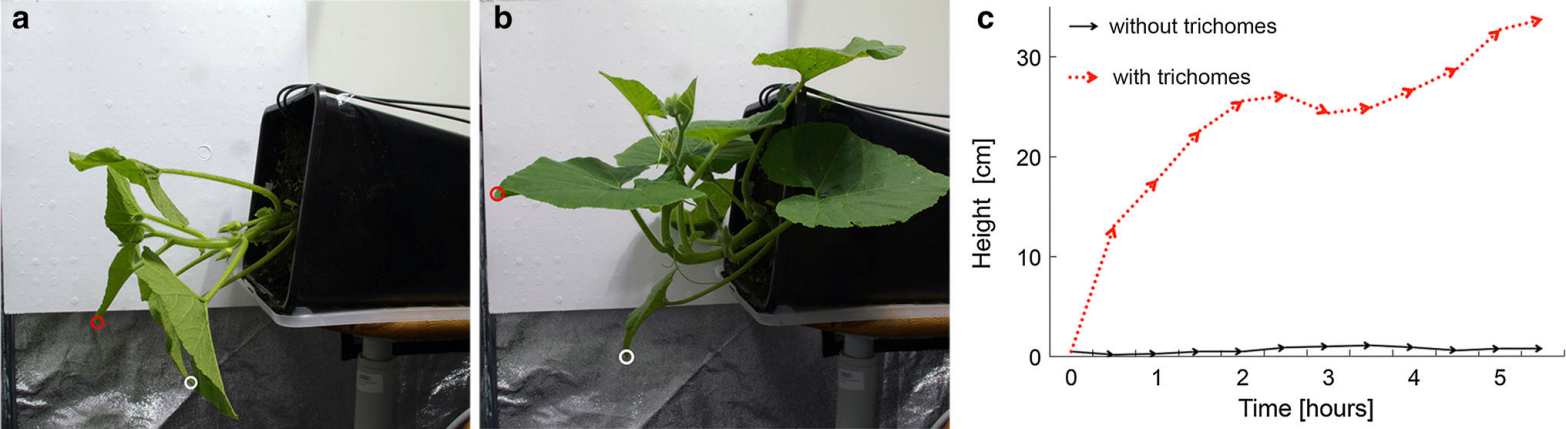
Reorientation of leaves in horizontally placed *C. maxima* ‘Bambino’ plants. **a** The initial phase (0 h) of the reorientation of the leaf with petioles with or without trichomes. Point trajectory for the analyzed leaf with petioles deprived of trichomes (*white circle*), and the petiole with trichomes (*red circle*). **b** The final phase (5 h) of the experiment, representing the lack of tropic response for the petiole deprived of trichomes and tropic reorientation of the leaf with an intact petiole. **c** Comparison of the movement of leaves with petioles without (*black line*) and with (*red line*) trichomes, based on the analysis of a time-lapse movie using Tracker software

An analysis of the leaf movement during their reorientation owing to the petioles action showed that leaves with intact trichomes (variant 1) reached their final orientations toward the light source and the gravity vector after approximately 2 h. Removal of the trichomes from the petioles (variant 2) blocked their movement without leaves wilting. It is important to mention that in this case, almost no evident movement was observed (Fig. 5a–c; Video 1, Supplementary Material). Longitudinally injured petioles (variant 4) reoriented the leaves immediately, i.e., in the same manner as non-wounded petioles, until the plane of the leaf was perpendicular to the light vector (Video 2, Supplementary Material). Petioles with the trichomes dehydrated with a solution of salt (variant 3) reoriented leaves about two times slower than petioles with non-dehydrated trichomes (Video 3, Supplementary Material).

### Microscopic observations

**Fig. 6:**
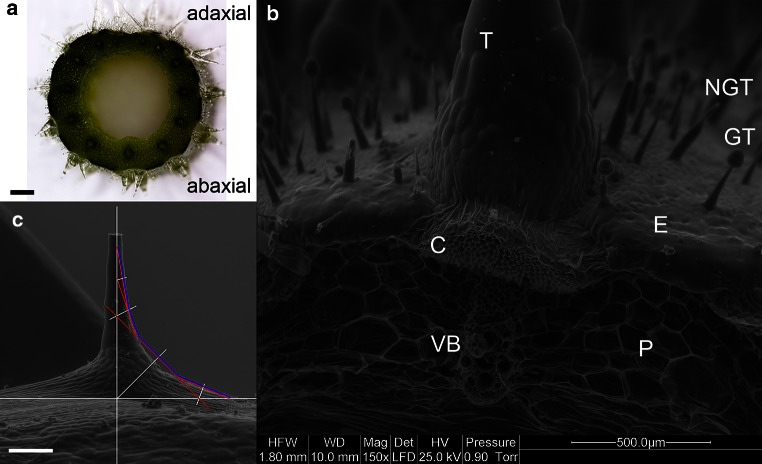
**a** Cross-section of the petiole with trichomes on the adaxial and abaxial sides. **b** Cross-section through the fragment on the petiole of the abaxial side. Multicellular non-glandular trichomes (*T*) on collenchyma (*C*) strand, above vascular bundle (*VB*), and ground parenchyma (*P*) are observed. In the epidermis (*E*), glandular trichomes (*GT*) and small non-glandular (*NGT*) trichomes can be seen. **c** The trichome from the abaxial side of the petiole with *plotted lines* of ‘*tensile triangles*’, with a *blue line* of optimal shape. Images by scanning electron microscopy (**b**–**c**). *Scale bar* 1.5 mm for (**a**) and 500 µm for (**b**–**c**)

With increased development of the petioles, a clear difference was observed between the trichomes on the adaxial surface and the trichomes on the abaxial surface in terms of shape (Fig. 6a). This observation is particularly important in the case of petioles that induced tropic reorientation of the leaves and did not grow vertically from the beginning. Trichomes located on the adaxial side of petioles are slender (Fig. 1a), similarly to those at the beginning of petiole growth. The trichomes of the abaxial epidermal surface of the petiole are massive, with a cone-shaped broad base (Fig. 1b). Trichomes from the adaxial side reached a maximum height of approximately *h*_max_ = 3.2 mm and a base diameter of approximately 500 µm. Trichomes from the abaxial side were shorter (*h*_max_ = 2.8 mm), with a base diameter of up to 1.4 mm. All the multicellular trichomes were positioned opposite to the collenchyma strands localized above the vascular bundles (Fig. 6a, b).

**Fig. 7:**
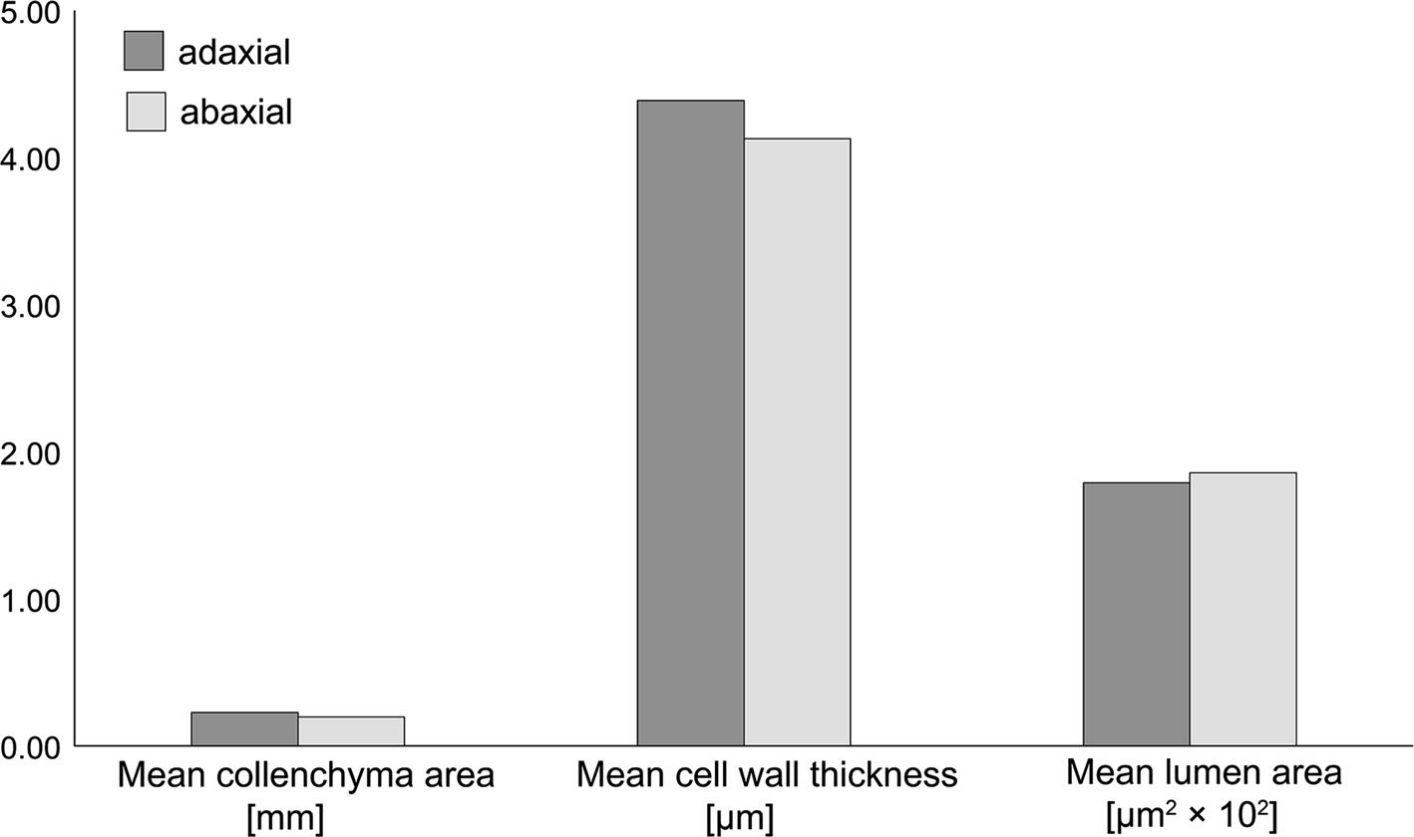
Comparison of the cross-section area of collenchyma strands, cell wall thickness and cell lumen surface area in collenchyma cells between the adaxial and abaxial sides of the petiole. Differences between means of 44 collenchyma strands from 11 petioles are not statistically significant

The method of ‘tensile triangles’ enabled the delimitation of the line of theoretical, optimized shape of the trichomes derived only from the abaxial side of the petioles (Fig. 6c). No significant difference in anatomical structure was observed between collenchyma on the adaxial and abaxial sides of the petiole. The mean cross-section surface area of the collenchyma strand amounted to 0.23 mm^2^ (SE ± 0.02) for the adaxial side and 0.20 mm^2^ (SE ± 0.02) for the abaxial side. Mean cell wall thickness amounted to 4.39 µm (SE ± 0.16) for the adaxial side and 4.13 µm (SE ± 0.14) for the abaxial side. Mean surface area of the cell lumen amounted to 186.01 µm^2^ (SE ± 14.8) for the adaxial side and 179.12 µm^2^ (SE ± 11.8) for the abaxial side (Fig. 7).

**Fig. 8:**
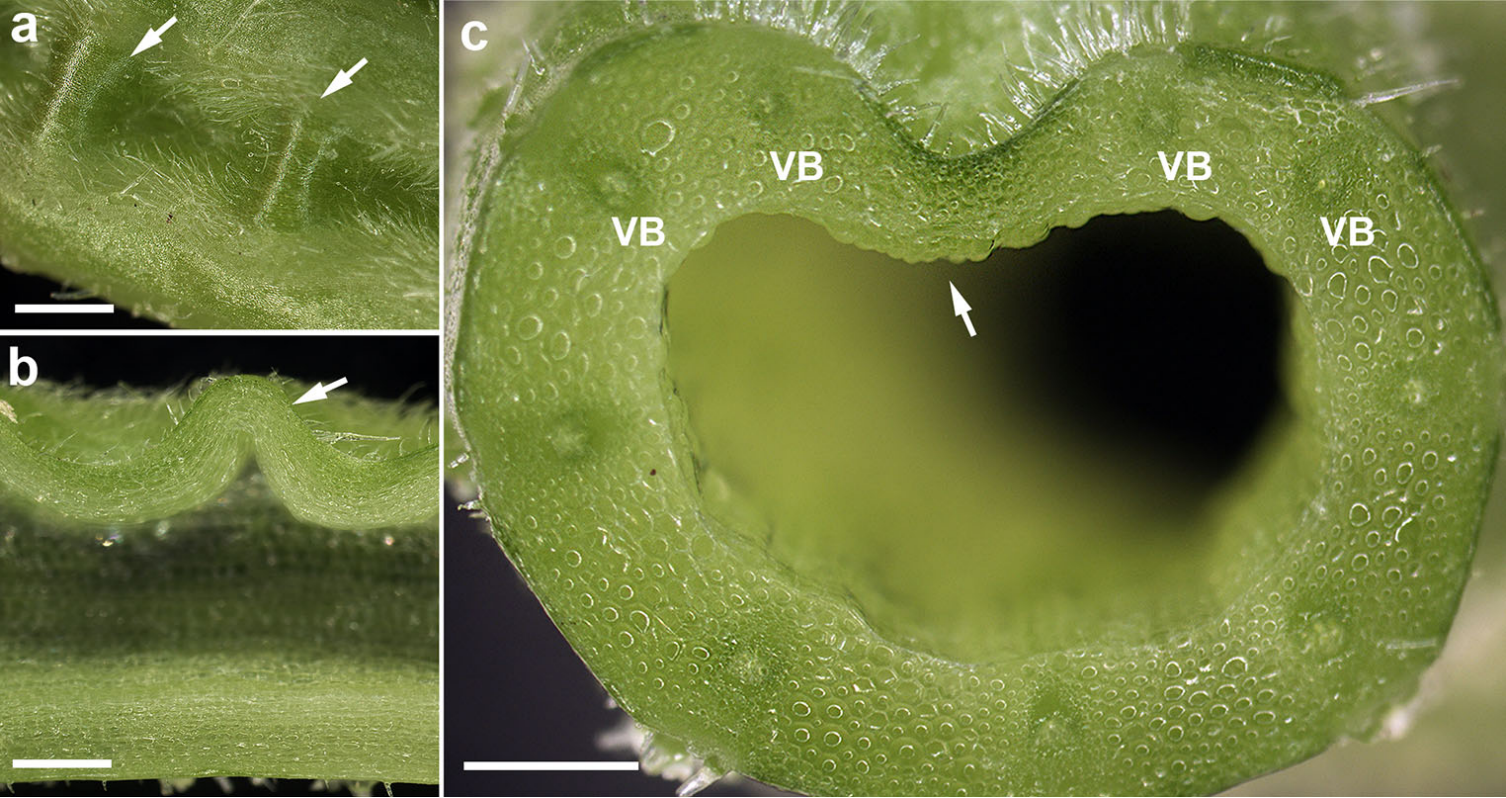
Local buckling on the adaxial side of the petiole. View from above epidermis (**a**) and longitudinal (**b**) and transverse (**c**) petiole sections. Buckling in the form of deep longitudinal *wave*-like grooves occurs in the tissue region where not vascular bundles (*VB*) are present. *White arrows* maxima of the *wave*-like pattern in (**a**) and (**b**), and the groove bottom in (**c**). *Scale bars* 1 mm

In some cases, when changes in the orientation of the leaves occurred, significant buckling was observed. However, such buckling was related only to the adaxial side of the petioles. It manifested itself as deep, wavy grooves along the strands where no collenchyma or vascular bundles were found (Fig. 8a–c).

### Mechanical tests

#### Breaking stress of collenchyma strands

**Fig. 9:**
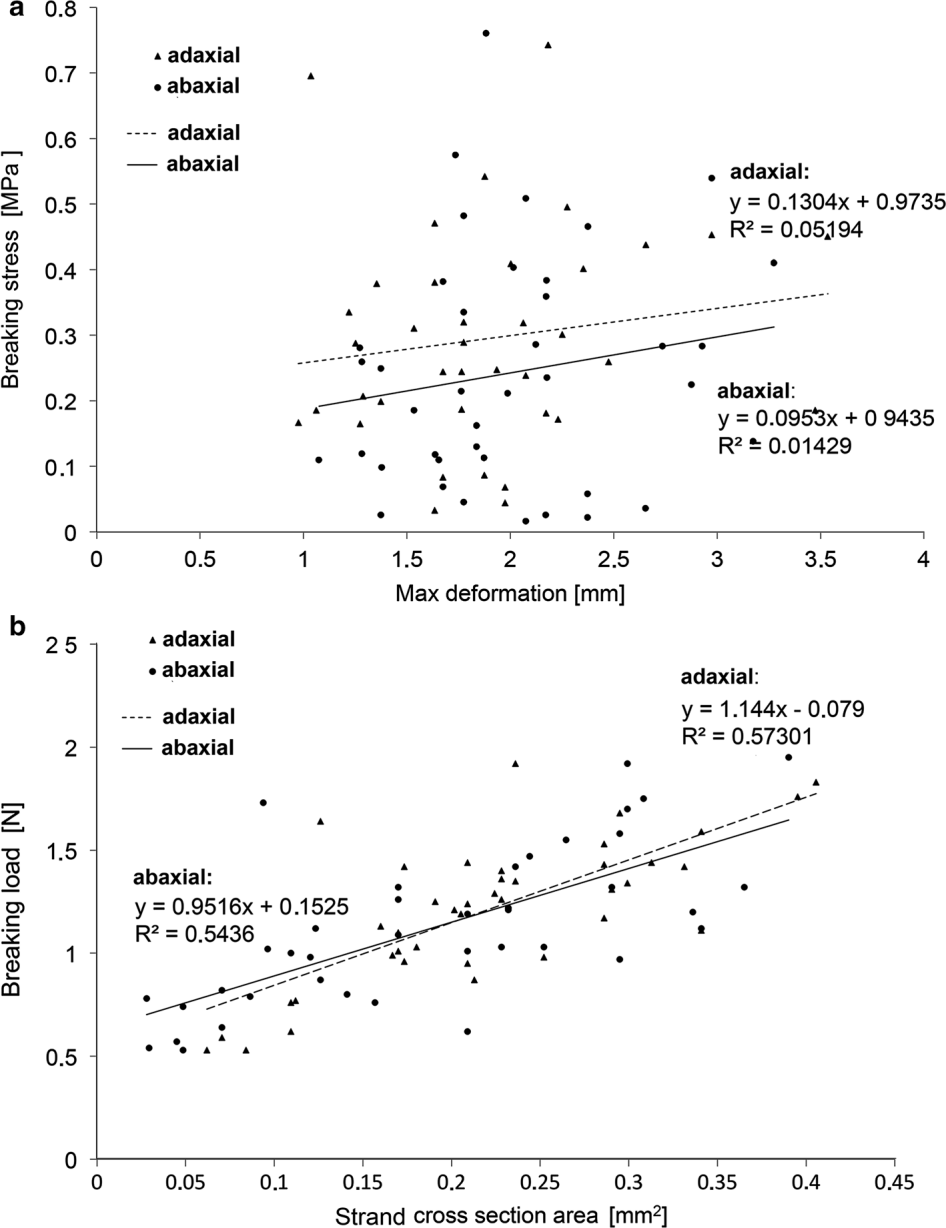
Results of the breaking tests of the isolated strands of collenchyma (10 cm long) with epidermis from adaxial (*triangle*) and abaxial (*circle*) sides of the petiole. **a** *Plot* presenting maximum deformation versus breaking stress. **b** *Plot* showing cross-section area of the strand versus maximum tension strength. The regression equation (*Y*) and coefficient of determination (*R* ^2^) are shown for adaxial (*dotted lines*) and abaxial (*solid lines*) sides of the petiole. Data for 7 leaves with 3 strands on the adaxial and 3 on abaxial side of each petiole

The results of the static tension tests of the 40 strands of collenchyma with epidermis revealed no clear relationship between the strength of the strands derived from the adaxial and abaxial sides of the petiole (Fig. 9a). The average breaking stress (BS_avg_) and maximum breaking stress (BS_max_) for strands from the adaxial side were BS_avg_ = 0.29 MPa (SD 0.16) and BS_max_ = 0.74 MPa. For strands from the abaxial side, the corresponding values were BS_avg_ = 0.25 MPa (SD 0.18) and BS_max_ = 0.76 MPa. In addition, there was also a weak correlation between maximum deformation and BS_max_. For the adaxial and abaxial sides, the correlation coefficients (*R*) were estimated to be 0.19 and 0.17, respectively. However, significant relationship was observed between the cross-sectional area of collenchyma strands and force inducing their rupture (Fig. 9b). For collenchyma derived from the adaxial and abaxial petiole surfaces, the correlation coefficient (*R*) for these features was 0.74 and 0.70, respectively.

**Fig. 10:**
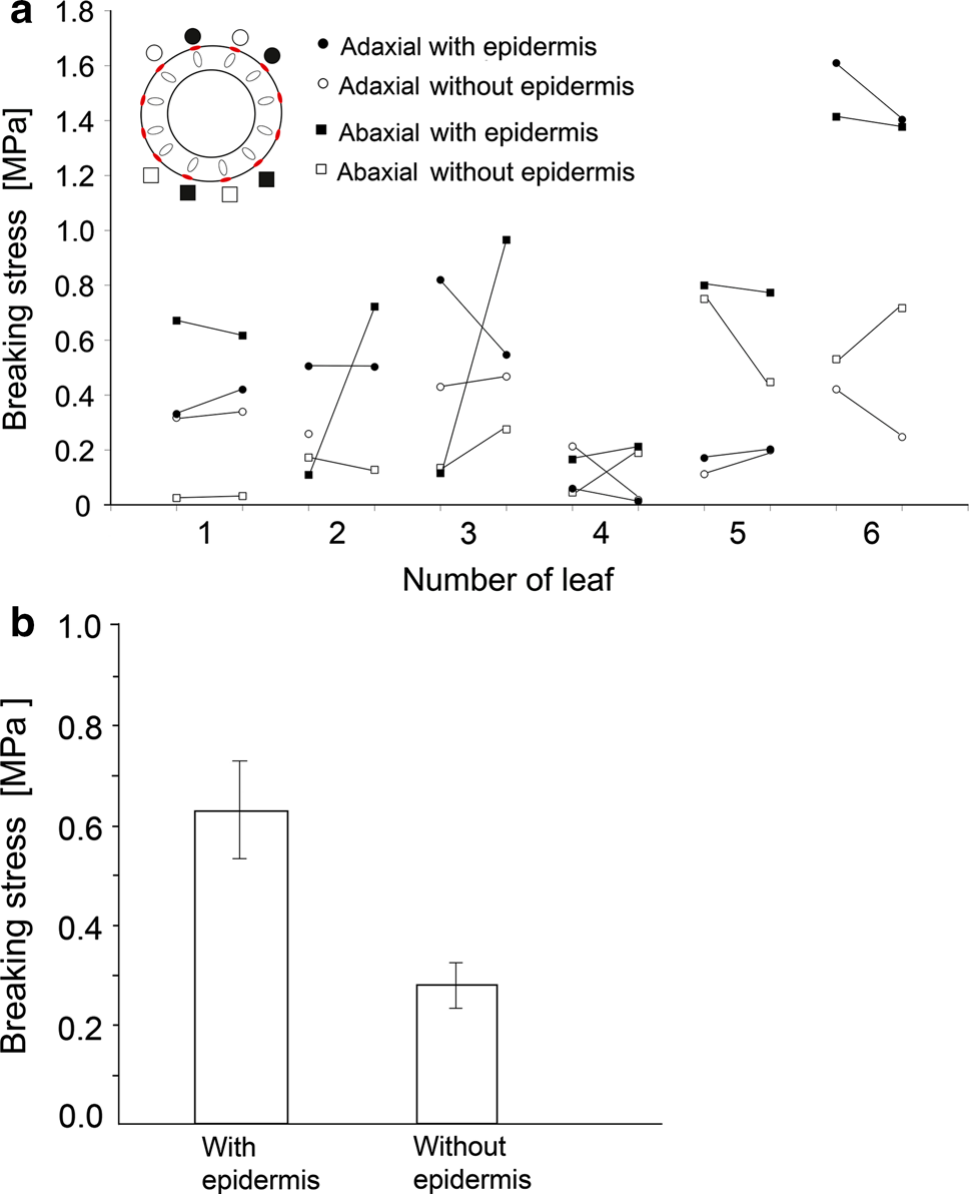
Results of the breaking test of isolated strands of collenchyma (10 cm long) with and without epidermis from adaxial (*circles*) and abaxial (*squares*) sides of the petiole; strands with intact epidermis (*black circles* and *squares*) and with removed epidermis (open *circles* and *squares*) are indicated. **a** Data for separate petioles from 6 leaves. **b** Average values for the petioles with and without epidermis are represented by two columns with *error bars* denoting ±SE (*n* = 24). Two collenchyma strands with epidermis and two strands without epidermis from adaxial and abaxial side of each petiole were tested

Twenty-three tensile strength tests were conducted on strands of collenchyma with the epidermis removed from 50 to 75 % surface of them. The results of 18 of the tests indicated lower resistance to tensile stress when compared to the neighboring strands of collenchyma with intact epidermises (Fig. 10a, b).

The tests of breaking stress of the collenchyma strands on adaxial and abaxial sides of five petioles revealed no significant effect of the vaseline coating. The average breaking stress (BS_avg)_ for strands from the adaxial side with epidermis without vaseline was 0.52 MPa (SE ± 0.07) and with vaseline was 0.58 MPa (SE ± 0.06); for strands without epidermis, the corresponding values were 0.26 MPa (SE ± 0.03) and 0.30 (SE ± 0.02). In the case of abaxial side of epidermis, the average breaking stress with epidermis without vaseline was 0.55 (SE ± 0.06) and with vaseline 0.60 (SE ± 0.05), for strands without epidermis the corresponding values were 0.29 (SE ± 0.03) and 0.33 (SE ± 0.03).

### Indentation

**Fig. 11:**
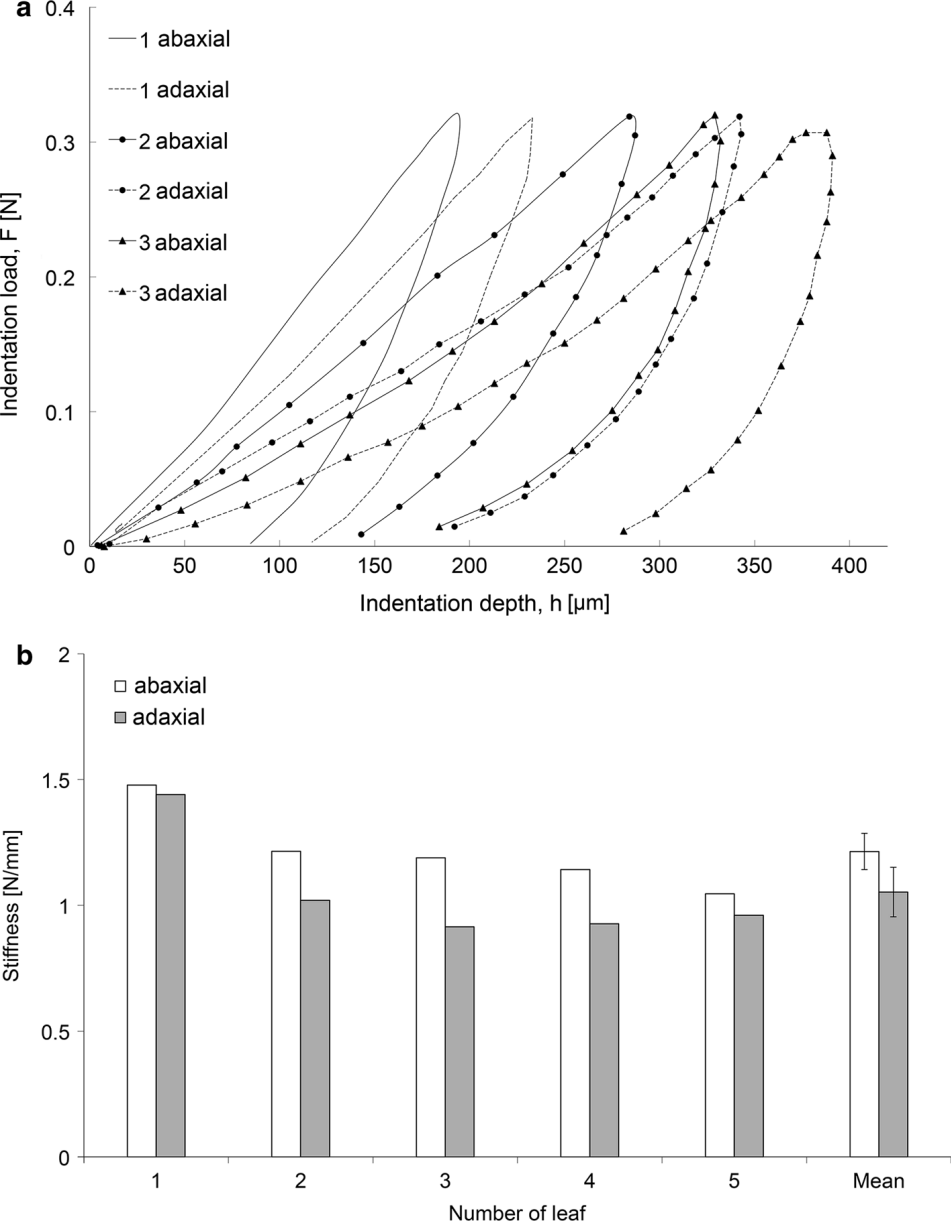
Results of the indentation test of the strands of collenchyma with epidermis not isolated from the surrounding tissues of ground parenchyma and vascular bundles. **a** Load-penetration (*P*–*h*) curves for one sample petiole (leaf no. 2). Measurements were conducted on samples (10 mm × 5 mm) from abaxial and adaxial sides obtained from three locations on the petiole: basal (*1*), middle (*2*), and apical (*3*). The experiment was repeated five times with similar results. **b** Average values of stiffness for the non-isolated strands on the adaxial and abaxial sides of the petioles in five individual leaves. The values of stiffness were calculated as the ratio of maximum force to maximum penetration for the three locations on the petioles. Mean values for the adaxial and abaxial sides for all of the five petioles are represented by the last two columns with *error bars* denoting ±SE (*n* = 5)

Results of the indentation tests revealed several general trends. For *P*–*h* curves measured on the adaxial and abaxial sides at three locations of the leaf petioles (basal, middle and apical), *S*_avg_ was greater in the case of samples cut from the abaxial side of the petiole than for the samples cut from the adaxial side. A sample plot for petiole no. 2 illustrating this relationship is shown in Fig. 11a. For the majority of the measured *P*–*h* curves, *S*_loc_ was also greater for the samples from the basal part of the petiole. On the basis of the results obtained for five leaves, we calculated average stiffness values as ratio of the maximum force to the maximum penetration for three locations on both sides of the petioles. For each of five leaves, the strands of collenchyma with epidermis in the abaxial side of the petiole were identified to be stiffer compared to those on the adaxial side (Fig. 11b). The mean values for the abaxial and adaxial sides of all the tested petioles differed significantly.

## Discussion

The obtained results indicate that removal of trichomes from petioles in *C. maxima* ‘Bambino’ blocks their tropic reaction. Analyzing the results, the question of whether the lack of response of leaf movement, when trichomes from the petioles are removed is the result of injury, can be raised. Wounding of herbaceous plants often causes leaf wilting and/or their browning (Woltering 1986; Lagrimini 1991; Ma et al. 2013). In our studies on *C. maxima* ‘Bambino’, no such effects were observed. In addition, the large longitudinal petiole wound had no influence on the leaf movement.

Such observations provoke many questions regarding the potential role of trichomes in the tropic movements of winter squash leaves. One of the most important questions addressed by this study is whether trichomes on the petiole play a role in the functioning or mechanical performance of the epidermis and collenchyma tissues. The reorientation of vigorously growing *C. maxima* ‘Bambino’ leaves is a biomechanical movement process during which a tissue-specific difference in the TS is likely to occur primarily in the epidermis–collenchyma strands between the adaxial and abaxial parts of the petiole. These strands showed no significant differences in terms of strand width, wall cell thickness, or collenchyma cell lumen.

According to some authors, herbaceous plant epidermis has been found in a high-stress state, resulting from the growth of inner tissues (i.e., the collenchyma), which means that their stress state is not subjected to turgidity regulation (Niklas and Paolillo 1997; Baskin and Jensen 2013). However, there is no evidence indicating the role of trichomes as a functional element of the epidermis and collenchyma, although in many species trichomes constitute a nearly integral part of this tissue. As it was shown in this work, the isolated collenchyma strands (with epidermis) from the adaxial and the abaxial sides of the petiole are not characterized by a difference in tension strength values. However, strands of collenchyma without epidermis were characterized by significantly lower strength. The results for the samples with exposed surfaces coated with vaseline indicate that the tension strength values were not significantly affected by the loss of turgor during the test. Nonetheless, the adaxial and abaxial sides of the petiole also exhibited no significant differences. However, the indentation of collenchyma strands not isolated from the surrounding petiole tissues indicates that the collenchyma on the abaxial side of the petiole was stiffer. It should be noted that in our experiments the tensile and indentation tests of collenchyma strands are mechanically distinct. The indentation test more likely measures average turgor, whereas the tensile test, rather, the cell wall strength. If we consider that the material strength of the walls is the same, the changes in TS revealed by indentation test are likely due to the turgor changes, which can be involved in the leaf petiole movement.

A question also arises whether the epidermis of *Cucurbita* petioles may be considered as a typical example of “tension stressed skin” (Kutschera and Niklas 2007), whereby even entire organs of herbaceous plants are viewed as “giant, pressurized protoplasts that are covered by a sturdy peripheral ‘organ wall’ with cuticle” (Kutschera 2008). In mechanical models, the epidermis is usually considered as a continuous external layer of a cylinder providing rigidness and being exposed to tensile stress due to pressure generated by turgid cells inside the cylinder. The growth of an organ depends on the changing elasticity of epidermis, which allows elongation of dividing cells in the core. However, in *Cucurbita* petioles, the cylinder is hollow, and the strands of collenchyma are located only above bicollateral vascular bundles. In this case, it might be suggested that the epidermis with multicellular trichomes is a tissue integrated with the hydrostatically active collenchyma, resulting in the formation of a single axial sector (vascular bundle-collenchyma–epidermis with trichomes) embedded in parenchyma cells. That is the reason why, despite a double longitudinal cut in the petiole, the epidermis lost its circumferential but not axial continuity. Perhaps, despite the major wound in the organ and severe local dehydration of the four cut surfaces, the longitudinal sectors (vascular bundle-collenchyma–epidermis with trichomes), affected by high pre-stress, could still function owing to changes in turgor pressure. This leads to the hypothesis that removal of the trichomes could relieve pre-stress in epidermis, preventing movement of the petiole. Dehydrating the trichomes with a solution of salt slowed down the movement of the petiole, but did not stop it. The plant likely began to hydrate the trichomes and equalize gradually hydrostatic pressure. In addition, local buckling (the Brazier effect) was not related to the abaxial side of the *C. maxima* ‘Bambino’ petiole, where large, multicellular trichomes are located. Although buckling in plants can be induced by growth (Green et al. 1996), it is known that this phenomenon often occurs because of a decrease in turgidity (Spatz et al. 1998). Thus, it seems probable that trichomes may be a kind of an additional hydrostatic pressure reservoir maintaining collenchyma and epidermis in a state of high tissue tension. In such a case, only a small change in the stress tissue gradient in the petiole can result in bending. Removal of the apical parts of trichomes may stimulate the relaxation of the existing hydrostatic pressure, causing a state of pre-stress for the entire system; such is the case of the epidermis and collenchyma.

In a study conducted by Hejnowicz and Sievers (1996), these authors observed that TS on the lower side of horizontally positioned stems of *Reynoutria* relaxed at the beginning of gravitropic movement; this effect was first observed in collenchyma. Our indentation tests of the strands of collenchyma that were not isolated from the remaining tissues of the petiole revealed an additional, very interesting property. After completion of the reorientation, the abaxial side of the petioles became more rigid. It might be indicated that movement of the petiole during leaf reorientation and the maintenance of its orientation in the gravitational field may be associated with TS changes after the completion of reorientation.

The morphology of trichomes in *C. maxima* ‘Bambino’ petioles is variable and the changes in their shape probably depend on different growth rates of the various zones of the petiole. In particular, trichomes from the abaxial side of the petiole change shape throughout their lifetime and form a broad, multicellular base. Cherdantsev and Grigor’eva (2010) found that the factor inducing mitotic divisions in the trichomes of *Draba*, providing a specific complexity of structure, is an increase in the surface area of the cells forming the base of the trichomes and causing a different distribution of stress within the cytoskeleton. Similar reactions may have also occurred in our experiments. It can be assumed that, due to a lack of lignified mechanical tissues, petioles, even after bending, must constantly retain the stress gradient and hence the variation in the structure of their trichomes. Moreover, it is possible that trichomes from the abaxial side of the petiole are subjected to higher stress. Thus, they modify shape to prevent local stress concentrations, as shown by the method of tensile triangles. Another phenomenon known as the propagation of cracks, which has been studied for years in the field of material engineering, is also interesting. It is known that a concentration of stress occurs in the crack tip and may be released by weak regions (e.g., lower strength materials or holes) in the front of the crack (Cook et al. 1964; Citarella et al. 2013). Trichomes on the strands of mechanically active tissues on the abaxial side of petioles may perhaps act as places in which stress release could occur, thereby preventing buckling and formation of cracks in the tissue. From this point of view, the form and shape of the trichomes might be a good indicator of stress in the epidermis. Owing to the relatively simple structure of trichomes, they may find wider applications in models of growth (Zhang and Oppenheimer 2004).

### *Author contribution statement*

UZ conceived and designed the research, conducted microscopic observations and time-lapse imaging, analyzed all data, and wrote the manuscript. SK and UZ conducted measurements and analyzed indentation test data. DG and UZ conducted breaking stress measurements. All authors read and approved the manuscript.

## Electronic supplementary material

Supplementary material 1 Video 1 Movement of *Cucurbita* *maxima* ‘Bambino’ leaves containing petioles with removed apical parts of trichomes (red arrows) and intact trichomes. The plants were placed perpendicular to the direction of gravity and the light source. The time-lapse imaging shows a reaction of two sample plants for periods of 22.5 h (samples no. 1 and 2) and one plant for 54 h (sample no. 3). Reorientation of the intact leaves is visible, and the leaves with the petioles with removed trichomes do not show a tropic response. Note that the removal of the trichomes from the petioles inhibited their movement but did not cause leaf wilting (MPG 17490 kb)

Supplementary material 2 Video 2 Movement of *Cucurbita maxima* ‘Bambino’ leaves with intact and longitudinally cut petioles (red arrows). The plants with intact trichomes were placed perpendicular to the direction of gravity and the light source. The time-lapse imaging shows a reaction of three sample leaf petioles for a period of 23, 30 and 16 h for samples no. 1, 2 and 3, respectively. Longitudinally cut petioles reoriented the leaf in the same manner as non-wounded petioles (MPG 21048 kb)

Supplementary material 3 Video 3 Movement of *Cucurbita maxima* ‘Bambino’ leaves with trichomes treated with a solution of NaCl (red arrows). The plants were placed perpendicular to the direction of gravity and the light source. The time-lapse imaging shows a reaction of two sample leaf petioles for a period of 29 and 24 h for sample no. 1 and 2, respectively. Reorientation of the petiole with NaCl treatment (red arrow) was significantly slower compared to the one with intact trichomes (MPG 11496 kb)

## Abbreviation

TS: Tissue stress
